# GPCRs overexpression and impaired fMLP-induced functions in neutrophils from chronic kidney disease patients

**DOI:** 10.3389/fimmu.2024.1387566

**Published:** 2024-08-26

**Authors:** Pablo Scharf, Silvana Sandri, Felipe Rizzetto, Luana Filippi Xavier, Daniela Grosso, Rebeca D. Correia-Silva, Pedro S. Farsky, Cristiane D. Gil, Sandra Helena Poliselli Farsky

**Affiliations:** ^1^ Department of Clinical and Toxicological Analyses, School of Pharmaceutical Sciences, University of São Paulo, São Paulo, Brazil; ^2^ Lagoa Federal Hospital, Rio de Janeiro, Rio de Janeiro, Brazil; ^3^ Galileo Biotech, Rio de Janeiro, Rio de Janeiro, Brazil; ^4^ Department of Morphology and Genetics, Federal University of São Paulo, São Paulo, São Paulo, Brazil; ^5^ Dante Pazzanese Institute of Cardiology of Sao Paulo, São Paulo, São Paulo, Brazil

**Keywords:** neutrophil-to-lymphocyte ratio, CXCR4, FPR, Annexin A1, fMLP

## Abstract

**Introduction:**

G-protein coupled receptors (GPCRs) expressed on neutrophils regulate their mobilization from the bone marrow into the blood, their half-live in the circulation, and their pro- and anti-inflammatory activities during inflammation. Chronic kidney disease (CKD) is associated with systemic inflammatory responses, and neutrophilia is a hallmark of CKD onset and progression. Nonetheless, the role of neutrophils in CKD is currently unclear.

**Methods:**

Blood and renal tissue were collected from non-dialysis CKD (grade 3 - 5) patients to evaluate GPCR neutrophil expressions and functions in CKD development.

**Results:**

CKD patients presented a higher blood neutrophil-to-lymphocyte ratio (NLR), which was inversely correlated with the glomerular filtration rate (eGFR). A higher frequency of neutrophils expressing the senescent GPCR receptor (CXCR4) and activation markers (CD18^+^CD11b^+^CD62L^+^) was detected in CKD patients. Moreover, CKD neutrophils expressed higher amounts of GPCR formyl peptide receptors (FPR) 1 and 2, known as neutrophil pro- and anti-inflammatory receptors, respectively. Cytoskeletal organization, migration, and production of reactive oxygen species (ROS) by CKD neutrophils were impaired in response to the FPR1 agonist (fMLP), despite the higher expression of FPR1. In addition, CKD neutrophils presented enhanced intracellular, but reduced membrane expression of the protein Annexin A1 (AnxA1), and an impaired ability to secrete it into the extracellular compartment. Secreted and phosphorylated AnxA1 is a recognized ligand of FPR2, pivotal in anti-inflammatory and efferocytosis effects. CKD renal tissue presented a low number of neutrophils, which were AnxA1^+^.

**Conclusion:**

Together, these data highlight that CKD neutrophils overexpress GPCRs, which may contribute to an unbalanced aging process in the circulation, migration into inflamed tissues, and efferocytosis.

## 1 Introduction

Neutrophil homeostasis is an essential and multi-orchestrated process for host immune surveillance. In a steady state, approximately 10^11^ neutrophils are produced daily in the bone marrow and subsequently released into the circulation. Neutrophils have a short lifespan in the blood, around 7–10 hours in humans (1). Mature neutrophils in the bone marrow or circulation constitutively express membrane receptors that respond to chemical mediators to control their trafficking within body compartments and to prompt a response to aggression. Therefore, fine-tuned control and synchrony occur between the bone marrow, blood, and clearance tissues to maintain neutrophil homeostasis and support host defense (2, 3).

G-protein coupled receptors (GPCRs) consist of seven α-helical transmembrane regions, and their downstream signaling relies on the activation of heterotrimeric G proteins (4). GPCRs play a crucial role in a variety of physiological processes, and malfunctioning of their signaling is implicated in multiple diseases. Consequently, GPCR agonists and antagonists serve as important pharmaceutical tools to modulate inflammatory diseases (4, 5).

Production, recruitment, and activation of neutrophils rely on GPCR expression and activation (4). Fine-tuned GPCR activation orchestrates the release of mature bone marrow neutrophils into the circulation and their half-life. The binding of CXCR4 (G protein-coupled chemokine receptor) to stromal cell-derived factor 1 (SDF-1), also known as CXCL12 (chemokine-CX-C motif ligand 12), retains mature neutrophils in the bone marrow. During neutrophil delivery into the blood, the downregulation of CXCR4 and the upregulation of CXCR2 occur in response to CXCL2 (chemokine-CX-C motif ligand 2). Then, circulating aged neutrophils downregulate CXCR2 and upregulate CXCR4 to home back into the bone marrow in response to SDF-1 (6, 7). Moreover, GPCR formyl peptide receptors (FPRs) contribute to granulopoiesis (8–10) and are pivotal receptors in the pro- and anti-inflammatory actions of neutrophils during the host response. A vast range of ligands can activate FPR1, including peptides derived from invading pathogens or mitochondria from dead cells, leading to downstream intracellular pathways associated with cytoskeletal reorganization, adhesion, chemotaxis, degranulation, NET formation, and NADPH oxidase activation (11). Conversely, the activation of FPR2 by endogenous peptides and proteins, such as lipoxin A4 and Annexin A1 (AnxA1), triggers effects related to the resolution of inflammation, such as efferocytosis and tissue repair (12–15).

Systemic inflammation is a hallmark of chronic kidney disease (CKD), triggered by uremic toxins and mediators secreted by the renal epithelium and infiltrating leukocytes (16, 17). Cohort studies have linked blood biomarkers of acute inflammation, including peripheral neutrophil counts, with the progression of renal dysfunction and failure (17). Indeed, a high neutrophil-to-lymphocyte ratio (NLR) is a marker of CKD, associated with the disease’s severity and progression (18–20). Nonetheless, the role of neutrophils in renal lesion is unclear. Evidence supports that CKD peripheral neutrophils are activated cells, producing reactive oxygen species (ROS), releasing granular and nuclear contents, and secreting pro-inflammatory cytokines (16, 21, 22). These actions damage the vessels and worsen cardiovascular complications and diabetes, which are the main underlying diseases in CKD (21) (23). Pro-inflammatory neutrophils also induce renal damage and the development of fibrosis detected in the final stage of kidney disease (21, 22). Moreover, CKD circulating neutrophils have been described as primed and unresponsive cells, which are associated with impaired host defense, supporting the increased risk of infections found in CKD patients (24–27).

Here, we used blood and renal tissue from non-dialysis CKD (grades 3–5) patients to evaluate the profile of GPCR expression in neutrophils and GPCR-mediated functions. As expected, CKD patients presented a high neutrophil-to-lymphocyte ratio (NLR) correlated with the progression of the disease; nonetheless, neutrophils were not abundant in the CKD renal tissue. This may be reflected by the overexpression and reduced activation of GPCRs involved in neutrophil aging, chemotaxis, and tissue repair actions.

## 2 Materials and methods

### 2.1 Study design

Adults with CKD stages 3 to 5 were enrolled by informed consent from nephrology outpatient clinics at Lagoa Federal Hospital (Rio de Janeiro, Brazil) and at Dante Pazzanese Institute of Cardiology of Sao Paulo (São Paulo, Brazil). Inclusion criteria for the CKD 1–5 cohort were: (a) Age ≥ 18 years, (b) Diagnosis of CKD according to the criteria of the National Kidney Foundation (NFK) -NKF-KDOQI (2002) and NFK-KDIGO (2013) (28, 29), and clinical manifestations, (c) Not currently receiving chronic hemodialysis or peritoneal dialysis, (d) Not the recipient of a kidney transplant, (e) Not known to be positive for human immunodeficiency virus, hepatitis B virus or hepatitis C virus, (f) Not currently receiving immunosuppressive therapy.

Healthy volunteers referred as control (employees and professionals from the hospital itself) were invited and include in the control group of our study after accepting informed consent. Inclusion criteria for the no CKD cohort were as follows: (a) Age ≥ 18 years, (b) No active medical conditions, (c) Not taking any medication (over the counter or prescription) within 48 h of study (excluding contraceptives), (d) No current or previous history of kidney disease.

Clinical and medicines data were obtained from medical records maintained in a secure, password-protected, web-based clinical database by Hospital e-SUS, the SUS IT Department - DATASUS hospital management system.

### 2.2 Sample collection

To analyze leukogram and serum parameters, venous blood (3–5 mL) was sampled using metal-free Safety Vacutainer blood collecting tubes containing >1.5 mg K2EDTA obtained from Becton Dickinson (New Jersey, USA). For glucose measurement, BD Vacutainer Fluoride/EDTA tubes were used. For neutrophil isolation, blood was collected using Safety Vacutainer blood collecting tubes with spray-dried sodium heparin from Becton Dickinson. Plasma was separated by centrifugation, recovered, aliquoted for measuring biochemical parameters, and stored at −80°C until AnxA1 serum levels measurement.

### 2.3 Hemogram and serum parameter analysis

The hemogram analyses were performed using the Sysmex XN-10–51100 automated equipment (Sysmex, Illinois, USA). Urea, creatinine, glucose, albumin, uric acid, homocysteine, and cortisol levels were measured by the AU680 Chemistry Analyzer (Beckman Coulter Diagnostics, California, USA). Potassium, sodium, and bicarbonate were determined using the ABL800 FLEX blood gas analyzer (Radiometer America, California, USA). The levels of human AnxA1 in serum and neutrophil supernatant were quantified using a commercial kit from MyBiosource (California, USA), according to the supplier’s instructions. The estimated glomerular filtration rate (eGFR) was calculated using the CKD-EPI formula based on the Chronic Kidney Disease Epidemiology Collaboration according to NKF-KDOQI (2002) and NFK-KDIGO (2013) guidelines. The neutrophil-to-lymphocyte ratio (NLR) was calculated by the ratio between the number of circulating neutrophils and lymphocytes counted.

### 2.4 Neutrophils isolation

Following plasma separation, the pellet containing leukocytes and erythrocytes was diluted with 0.9% NaCl (saline solution) at a 1:1 ratio and submitted to density gradient centrifugation using Histopaque (1077; Sigma Aldrich, Missouri, USA) as previously described. Mononuclear cells were then discarded, and the cell pellet containing erythrocytes and neutrophils was incubated with 3% Dextran (Sigma Aldrich) diluted in saline on ice for 30 minutes. Subsequently, the neutrophil-enriched supernatant was recovered and submitted to erythrocyte lysis. The cells were incubated with an ammonium chloride buffer at a 1:9 ratio for five minutes on ice, followed by centrifugation. This procedure was repeated until complete erythrocyte lysis was achieved. The neutrophils were then counted using a Neubauer chamber. For flow cytometry analysis, neutrophils were fixed in 2% paraformaldehyde (PFA), washed, resuspended in 1% bovine serum albumin (BSA; Gibco, Massachusetts, USA) in phosphate-buffered saline (PBS), and stored at 4°C until analysis. Fresh neutrophils were submitted to ex-vivo analysis, as described in the following sections. All buffers and solutions were prepared using salts acquired from Synth (São Paulo, Brazil).

### 2.5 Flow cytometry analysis

Neutrophils were characterized using specific antibodies for membrane and intracellular markers. For membrane markers, cells fixed with 2% PFA were washed with PBS containing 0.1M glycine, followed by washing with 1% BSA in PBS (BSA/PBS), and incubated with fluorophore-conjugated anti-human antibodies: CD18 (1:50; Southern Biotechnology Associates, Alabama, USA), CD11b (1:50; BD Pharmingen, New Jersey, USA), CD62L (1:100; Southern Biotechnology Associates), CXCR4 (1:200 PE/Cyanine 5; BioLegend, California, USA), CXCR2 (1:50; R&D Systems, Minnesota, USA), FPR1 (1:100; Bioss, Boston, MA, USA), human FPRL1/FPR2 (1:100; BD Biosciences, Minneapolis, MN, USA), and CD66b (1:75; BD Pharmingen) for 40 minutes in the dark at room temperature. The AnxA1 expression was assessed intracellularly by a permeabilization procedure using 0.1% Triton X-100 (Sigma-Aldrich) in PBS (15 minutes at room temperature) and at the membrane in non-permeabilized cells. For AnxA1 labeling, cells were washed once with PBS containing 0.1M glycine, twice with 1% BSA in PBS (BSA/PBS), and incubated with primary anti-human AnxA1 antibody (1:100; Invitrogen, Massachusetts, USA) for 1 hour at 37°C. Next, cells were washed twice with BSA/PBS and incubated with a secondary goat anti-rabbit antibody conjugated to Alexa Fluor 488 (1:500; Invitrogen) for 50 minutes in the dark at room temperature. Apoptosis was measured using Annexin V-FICT (1:100; Invitrogen) diluted in a binding buffer and incubated for 20 minutes in the dark at room temperature, followed by a single wash with PBS. The cells were analyzed using a BD Accuri™ C6 flow cytometer (BD Biosciences, New Jersey, USA), recording 10,000 or 50,000 events, and using BD CSampler™ Analysis software (version: 1.0.2641–21, BD Biosciences). Data were extracted using the gating strategy reported in **Supplementary Figure 1** and reported as the frequency (%) or the expression by detecting median fluorescent intensity (MFI).

### 2.6 Boyden chamber assay

Neutrophils (2.5 × 10^6^/ml) from control or CKD donors were resuspended in 30 μL Hanks balanced salt solution (HBSS) supplemented with 0.05% BSA and transferred to the upper compartment of a ChemoTx-101–8 microplate (Neuro Probe, Inc., Maryland, USA) (30). The bottom part of the microplate was filled with 300 μL of HBSS containing 50 or 100 nM N-formyl-methionyl-leucyl-phenylalanine (fMLP; Sigma Aldrich). The microplate was incubated under controlled conditions (37°C, 5% CO_2_) for 2 hours. Cell numbers in the bottom compartments were obtained via a hemocytometer and optical microscope. The assay was performed in triplicate.

### 2.7 Actin polymerization assay

Neutrophils from both control and CKD donors were used to determine actin polymerization through a flow cytometry assay. Cells were stimulated with 100 nM fMLP in RPMI supplemented with 10% heat-inactivated fetal bovine serum (FBS; Gibco, Massachusetts, USA) and 1% streptomycin/penicillin solution (Gibco) for 0.5, 1, and 3 minutes. The cells were then fixed with 100 μL 2% PFA buffered and incubated for 25 minutes at room temperature. After that, they were washed twice with BSA/PBS and permeabilized with 1 mL BD CytoFix/Wash buffer (BD Biosciences). Intracellular staining was done using FITC-phalloidin (1:100; Invitrogen) for 30 minutes at 37°C, followed by another wash with PBS. Finally, 30,000 events were acquired in a BD Accuri™ C6 flow cytometer. The data obtained were represented as MFI.

### 2.8 ROS measurement

Neutrophils from control and CKD donors were incubated in the absence and presence of 50 or 100 nM fMLP in complete Roswell Park Memorial Institute (RPMI) 1640 Medium (Gibco) for 30 minutes at 37°C. Subsequently, ROS production was measured by incubating neutrophils with 10 µM 2’,7’-dichlorofluorescin diacetate (DCFH-DA; Invitrogen). A total of 30,000 events were acquired using an Accuri C6 flow cytometer. The data obtained were represented as mean fluorescence intensity (MFI).

### 2.9 Apoptotic neutrophils

To induce neutrophil apoptosis, 5 × 10^5^ cells from control and CKD donors were seeded in a 96-well plate (U-bottom; Corning, New York, USA) and incubated in serum-starved Dulbecco’s Modified Eagle Medium/Nutrient Mixture F-12 (DMEM/F12) medium (Gibco) for 18 hours. After incubation, apoptosis was measured by flow cytometry, and the free-cell supernatant recovered after centrifugation was stored at −80°C until AnxA1 levels measurement.

### 2.10 Biopsies

The study used biopsy samples from five CKD patients undergoing kidney cancer investigation submitted to partial or total nephrectomy. Among them, 4 patients were diagnosed with clear cell renal adenocarcinoma, and one with papillary-type renal cell carcinoma at different stages as described in the Supplementary Material. The analyzed regions were taken from tumor-free areas.

#### 2.10.1 Tissue processing and staining

Kidney tissue was fixed with 10% buffered formalin at 4°C and histologically processed for paraffin embedding. Sections (5 μm) were stained with hematoxylin and eosin for morphological investigation. Periodic Acid-Schiff (PAS) staining was performed using a commercial kit following the manufacturer’s instructions (EasyPath; São Paulo, Brazil) to verify fibrosis.

#### 2.10.2 Immunofluorescence

Briefly, sections were permeabilized with 1% Triton-X-100 (Sigma-Aldrich) in PBS at 4°C for 15 minutes. Sections were then blocked with 5% BSA (Sigma Aldrich) in PBS for 30 minutes at room temperature. Patients’ samples were incubated with anti-AnxA1 goat antibody (1:100) (AF3770; R&D SYSTEM) and anti-myeloperoxidase rabbit antibody (1:50; Biodesign International, Inc., Maine, USA) overnight at 4°C, followed by incubation with anti-goat Alexa Fluor 467 and anti-rabbit Alexa Fluor 488 secondary antibodies (1:200; Invitrogen). Negative controls were obtained by omitting the primary antibody. Immunofluorescence was analyzed using a Leica TCS SP8 CARS confocal microscope, and images were captured using the LAS X Life Science software.

### 2.11 Statistical analysis

Data were analyzed using GraphPad Prism version 9 (GraphPad Software®, California, United States). Data normality was performed using the Kolmogorov-Smirnov test. Next, we employed the parametric tests Welch’s t-test or one-factor analysis of variance (One-Way ANOVA) followed by Tukey *post-hoc* tests when appropriate. Pearson’s correlation tests were performed for the correlation analysis. Differences between assessed means and correlation coefficients were considered significant assuming *p < 0.05*. All results are shown as mean ± standard error of the mean.

## 3 Results

### 3.1 General data of the patients

The study included 36 patients and 11 control participants for comparison. As shown in **Table 1**, all 36 patients had varying stages of chronic kidney disease (CKD), with 47.20% in stage 3, another 47.20% in stage 4, and the remaining 5.50% in stage 5. The estimated Glomerular Filtration Rate (eGFR) of the CKD patients was approximately four times lower than normal, and high levels of urea and creatinine were observed. Additionally, homocysteine levels were found to be higher, and serum bicarbonate levels were lower in these patients. Annexin A1 (AnxA1) levels were elevated in the blood of CKD patients compared to control donors. Out of the CKD patients, 40% were also diagnosed with Type II Diabetes Mellitus, while 11% were smokers and 5.5% were alcoholics. All CKD patients were receiving conservative treatment, including medication and nutritional management. The main types of medication used were statins, fibrates, thiazide diuretics, and antihyperglycemics, as shown in **Supplementary Table 1**.

**Table 1:** Clinical analysis of controls and CKD patients.

	Disease Stage		(n)	(%)		
		3	17	47,20%		
		4	17	47,20%		
		5	2	5,50%		
		**Control**		**CKD**		
Renal Function						
	Ref. Values	Average	min – max	Average	min – max	*p-value*
**eGFR (ml/min/1.73m^2^)**	60>	94,8 ± 20,84	66,4 - 133,7	29,84 ± 12,29	11,7 - 66,3	0,0001
Biochemical and Serum Analyses						
**U (mg/L)**	17 - 43	39,8 ± 2,61	37 – 45	75,6 ± 32,42	37 – 156	0,0001
**Cr (mg/L)**	0,84 -1,25	0,93 ± 0,17	0,7 – 1,1	2,2 ± 0,84	0,8 – 4,4	0,0001
**Glu (mg/L)**	70 - 99	89,3 ± 6,93	78 – 100	107,6 ± 46,4	64 – 323	0,2210
**K (mmol/L)**	3,5 – 5	4,25 ± 0,29	3,8 – 4,8	4,69 ± 0,56	3,4 – 5,8	0,2844
**HCO_3_ (mmol/L)**	21 – 27	27,85 ± 5,16	22,3 – 36,7	23,89 ± 3,66	18,2 – 32,5	0,0008
**Uric Acid (mg/L)**	2,4 – 7	5,6 ± 0,54	4,7 – 6,5	6,69 ± 3,36	3,2 – 9,6	0,0009
**Alb (g/dL)**	3.5 – 5.2	3,93 ± 0,28	3,4 – 4,2	4,14 ± 0,3	3,6 – 4,6	0,1633
**Hcys (µmol/L)**	5 – 15	7,66 ± 1,8	5,6 – 9,6	13,76 ± 3,16	8,7 – 19,7	0,0001
**Cortisol (mcg/dL)**	5,3 – 22,5	11,86 ± 2,03	8,9 – 12,7	12,56 ± 3,59	8,9 – 18,6	0,9955
**Annexin A1 (ng/mL)**	N/A	4,8 ± 3,7	0,58 – 11,58	9,42 ± 8,61	0,68 – 24,56	0,0435

### 3.2 High neutrophil count and NLR are associated with CKD progression

**Figure 1:**
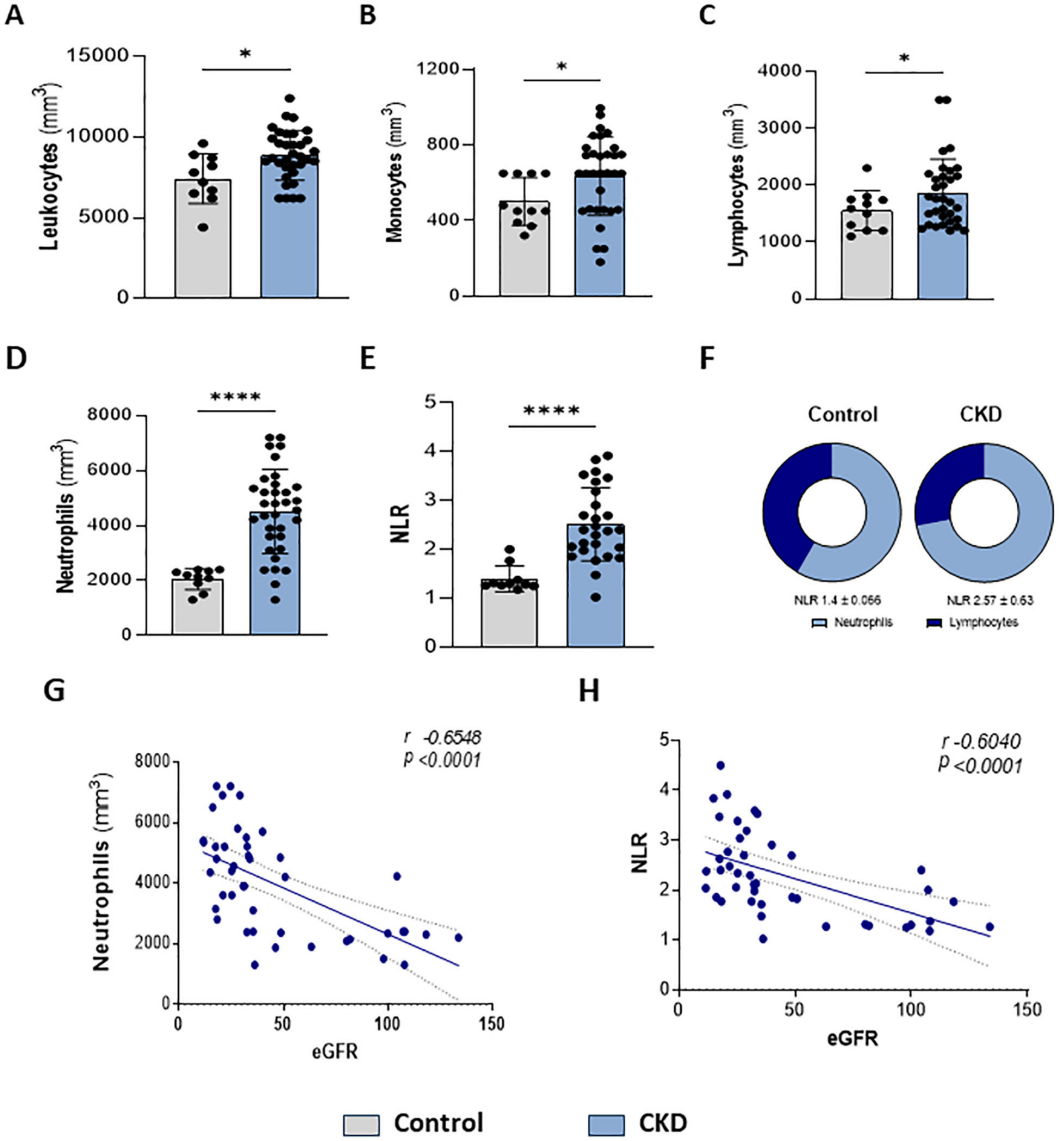
Higher neutrophil count is associated with renal dysfunction. Characterization of peripheral leukocytes. Count of total leukocytes **(A)**, monocytes **(B)**, lymphocytes **(C)**, and neutrophils **(D)**. Neutrophils-lymphocytes ratio (NLR) **(E–F)** (Control n= 11 and CKD n=36). Correlation of neutrophils **(G)** and NLR **(H)** with estimated Glomerular Filtration Rate (eGFR) *p<0.05; ****p<0.001 *vs* Control.

Here, we confirmed that CKD patients in stages 3–5 presented leukocytosis (**Figure 1A**). Monocytes (**Figure 1B**) and lymphocytes (**Figure 1C**) showed slight increases. Circulating neutrophils were notably elevated (**Figure 1D**). Since a high neutrophil-to-lymphocyte ratio (NLR) is a hallmark of CKD progression, herein NLR was increased (**Figures 1E, F**), which correlated with a decreased eGFR (**Figures 1G, H**).

### 3.3 CKD neutrophils are activated and senescent

**Figure 2:**
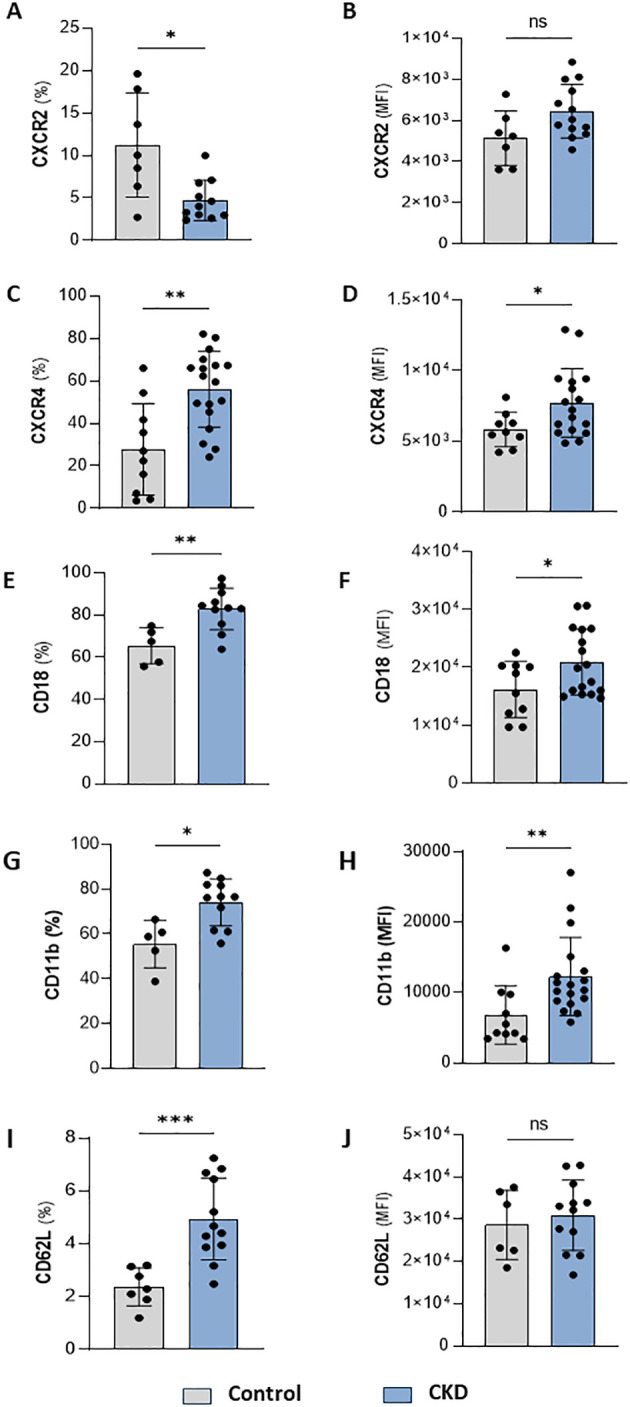
CKD neutrophils have heterogeneous phenotypes. Frequency of positive neutrophils and expression for CXCR2 **(A, B)**, CXCR4 **(C, D)**, CD18 **(E, F)**, CD11b **(G, H)** and CD62L **(I, J)** from control and CKD donors. (Control n= 4–10 and CKD n=10–18) *p<0.05; **p<0.01; ***p<0.001 vs Control. ns, Not significant.

Neutrophilia occurs due to enhanced production of neutrophils from the bone marrow and is associated with systemic inflammation. Infiltrated neutrophils can display an enhanced half-life, which can result from cellular activation or impaired clearance processes. Quantification of membrane proteins involved in cell maturation, senescence, adhesion, and migration point out alterations in the trafficking of neutrophils in the body compartments. Here, we found that CKD neutrophils display a heterogeneous phenotype of proteins related to activation, senescence, and mobilization. These cells were characterized by a lower frequency but higher expression of CXCR2 (**Figures 2A, B**) and a higher frequency and expression of CXCR4, CD18, CD11b, and CD62L (**Figures 2C–J**).

### 3.4 CKD neutrophils are hyporesponsive to FPR1 agonist

**Figure 3:**
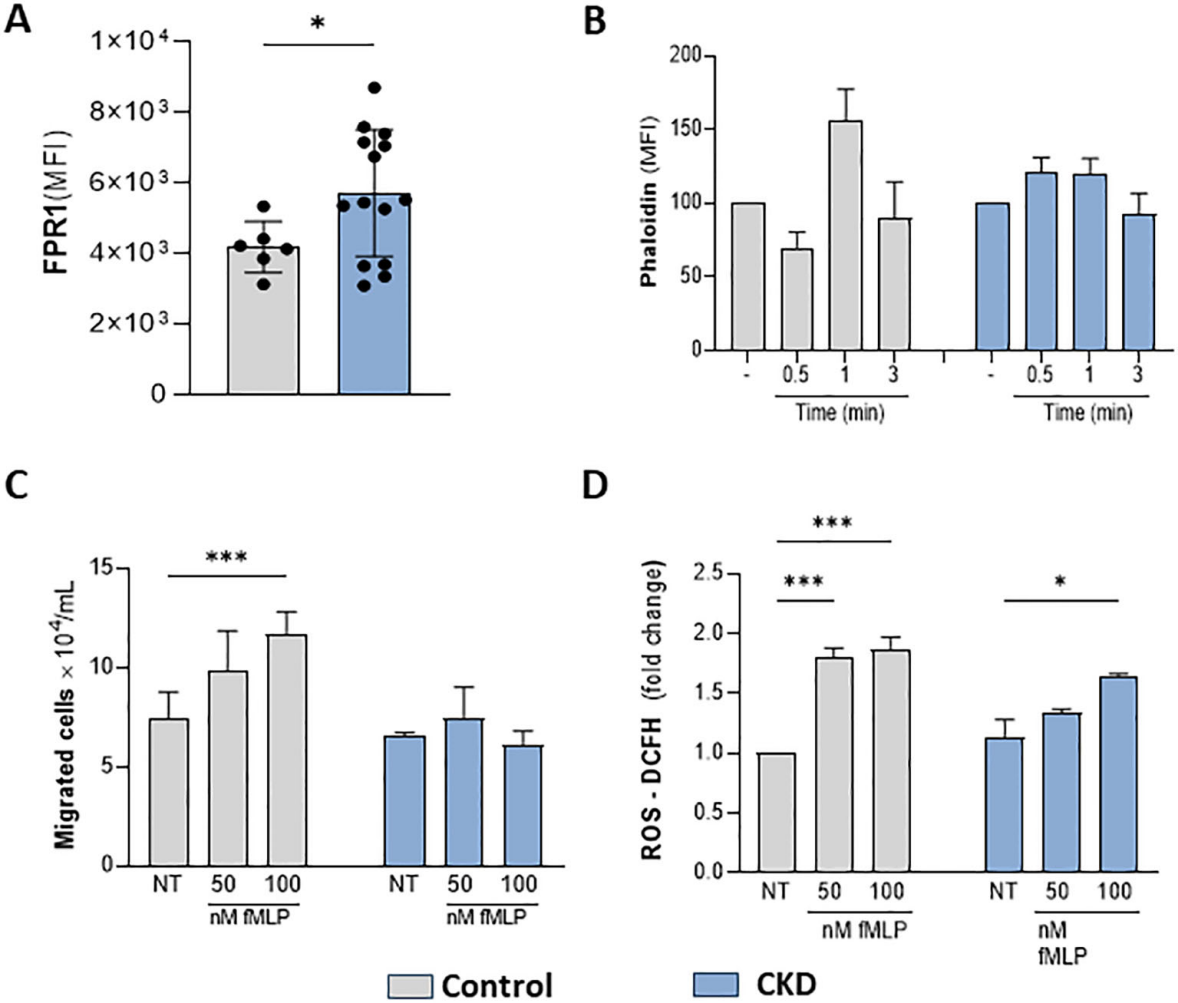
CKD neutrophils did not respond to the FPR1 agonist fMLP. FPR1 expression in neutrophils **(A)**. Chemotaxis **(B)**; Phalloidin expression **(C)** and ROS production **(D)** were decreased in CKD neutrophils stimulated with fMLP. (Control n= 3–5 and CKD n= 3–18). *p<0.05 *v*s Control or NT-control. ***p<0.001 *vs* NT- Control; *p<0.05 vs 50 nM fMLP-CKD vs 50 nM fMLP Control.

The effective response of neutrophils during host defense relies on GPCR chemosensors, which recognize a repertoire of plasma and inflammatory mediators. Here, we tested whether CKD neutrophils could respond to a specific FPR1 agonist. fMLP (N-Formyl-Met-Leu-Phe) is a formyl peptide derived from invading pathogens or mitochondria from dead host cells, binding to FPR1 to signal pro-inflammatory pathways in neutrophils. CKD neutrophils expressed higher levels of FPR1 than the control group (**Figure 3A**). However, in CKD neutrophils, fMLP did not induce phalloidin expression, chemotaxis, and ROS production (**Figures 3B–D**). Altogether, the data obtained here show that CKD neutrophils are hyporesponsive to FPR1-mediated actions.

### 3.5 Intracellular AnxA1 is overexpressed in CKD neutrophils but is not secreted

**Figure 4:**
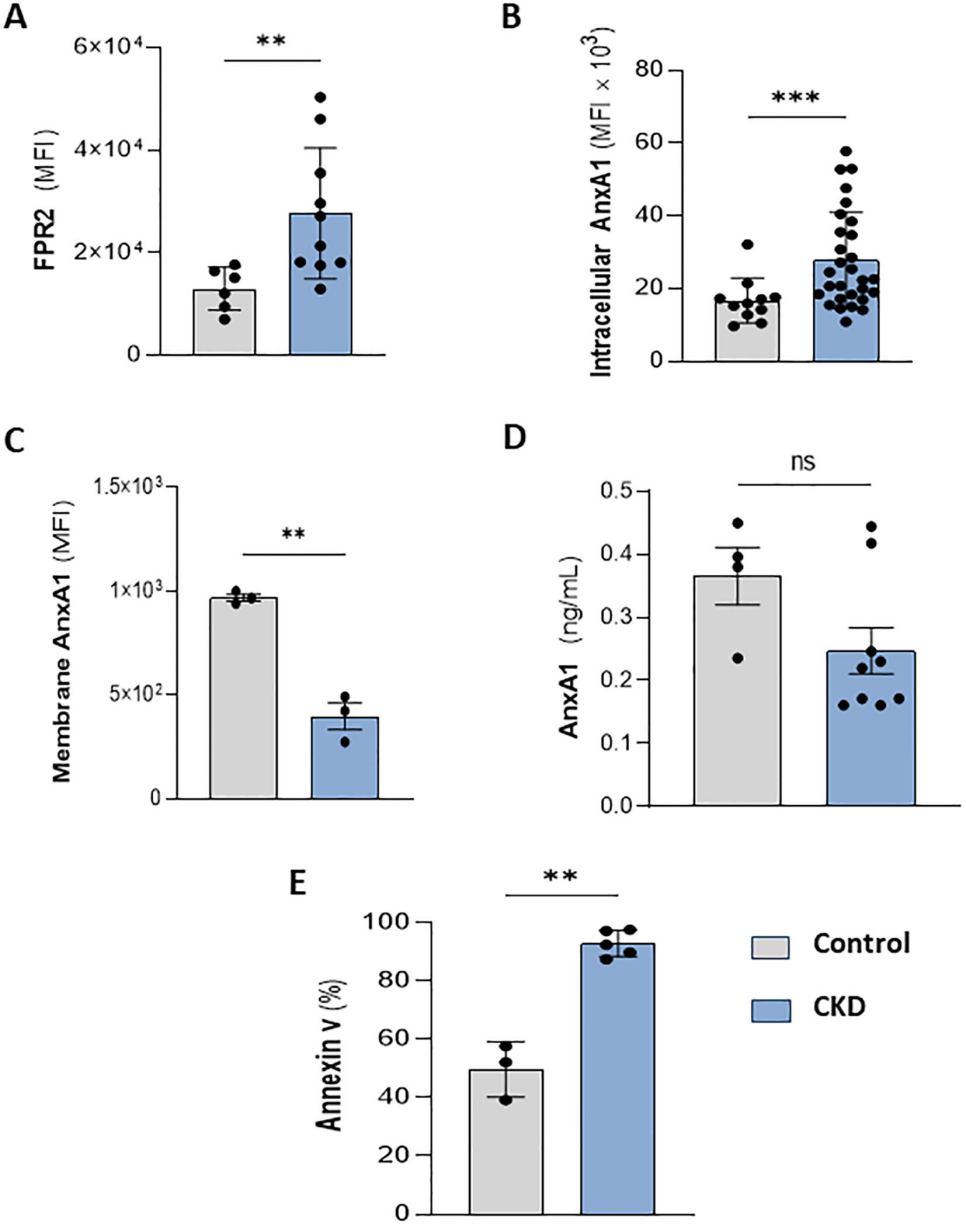
CKD neutrophils overexpress intracellular AnxA1. Increased FPR2 expression in CKD neutrophils **(A)**. AnxA1 expression in neutrophils at intracellular **(B)** and membrane compartments **(C)**. AnxA1 secretion from neutrophils **(D)** and apoptotic neutrophils **(E)** cultured for 18 hours under serum starvation conditions. (Control n= 3–11 and CKD n= 3–36) **p<0.01; ***p<0.001 CKD *vs* Control. ns, Not significant.

Meanwhile, FPR1 agonism by formyl peptides triggers pro-inflammatory effects in neutrophils. Conversely, the binding of endogenous peptides and proteins, such as Lipoxin A4 and AnxA1, to FPR2, expressed on neutrophil membranes, evokes anti-inflammatory and efferocytosis effects (13, 31, 32). Interestingly, CKD neutrophils also expressed higher levels of FPR2 (**Figure 4A**). Endogenous AnxA1 is secreted and phosphorylated to induce autocrine anti-inflammatory effects elicited by FPR2 activation (12). Here, we detected that CKD neutrophils contain higher levels of intracellular AnxA1 expression but lower levels of membrane-bound AnxA1 compared to the control group (**Figures 4B, C**). Although CKD neutrophils overexpress AnxA1, neutrophils incubated for 18 hours secrete similar levels of AnxA1 as the control group (**Figure 4D**), despite the high rate of apoptotic neutrophils (**Figure 4E**), which is a requirement for AnxA1 secretion during efferocytosis (33, 34).

### 3.6 Renal infiltrated neutrophils are AnxA1^+^

**Figure 5:**
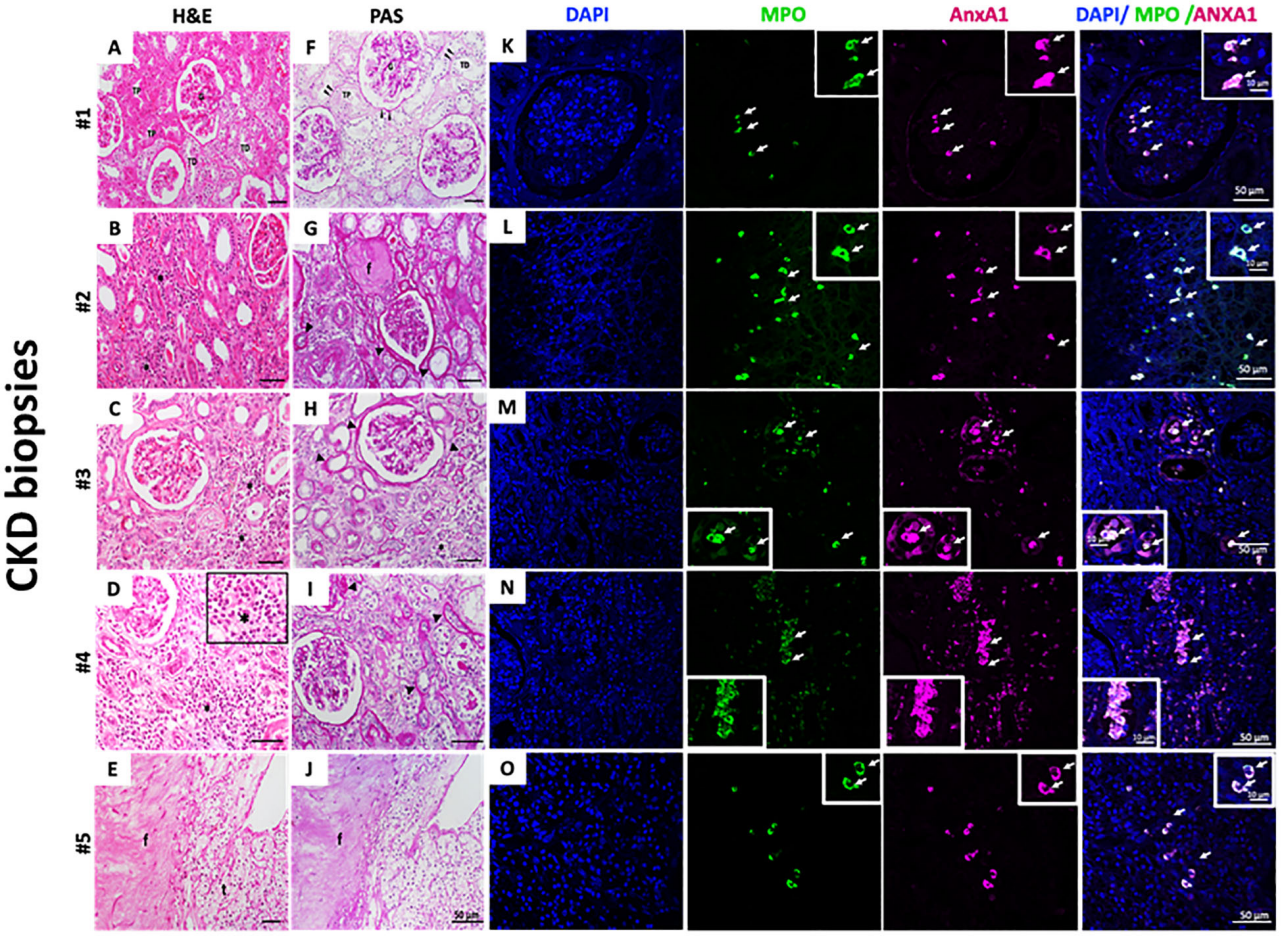
Infiltrate neutrophils in CKD kidney biopsies are AnxA^+^. H&E **(A–E)** and PAS staining **(F–J)**. Myeloperoxidase (MPO) and AnxA1 labeling **(K–O)**. Five CKD biopsies were selected and stained for morphological, fibrosis visualization, and immunofluorescence. **Figure 5D** – Insert: * immune cells infiltrated; MPO and AnxA1 labeling. The image is representative of at least three fields analyzed on each biopsy.

Selected biopsies from five patients with CKD (Cr 1.3 ± 0.12 mg/dL; eGFR 52.4 ± 8.08) were analyzed. As observed by H&E staining, the first patient’s sample showed normal tissue architecture characterized by intact glomeruli of the renal corpuscle (G); distal convoluted tubules (DTC); and proximal convoluted tubules (PCT) (**Figure 5A**). In **Figures 5B–E**, the samples presented CKD features such as interstitial nephritis characterized by the presence of inflammatory infiltrate in the renal interstitium (asterisks and insert - **Figure 5D**). A thickened basement membrane was detected in renal corpuscles and convoluted tubules (arrowheads - **Figure 5F**), as observed by PAS staining. Additionally, it highlights the basement membrane in the parietal layer of the renal corpuscle Bowman’s capsule and in the proximal and distal convoluted tubules (arrowheads - **Figures 5F-J**). Some regions showed tissue fibrosis (f) and tubules in the process of degeneration (t) (**Figures 5G, J**). By immunofluorescence, neutrophils were identified by myeloperoxidase (MPO) labeling. As observed in **Figures 5K–O**, neutrophils were found in all samples, and the number of infiltrated neutrophils varied among CKD biopsies. Further analysis showed that neutrophils were also labeled for AnxA1.

## 4 Discussion

Chronic Kidney Disease (CKD) is a global health burden, affecting 13.4% of the world ‘s population (35, 36). CKD progression leads to severe morbidity and high mortality, which can be attributed to impaired renal function during end-stage kidney disease (ESKD) or to cardiovascular diseases and secondary infections (36, 37). Neutrophils contribution to CKD and related diseases development is an inconclusive subject, despite high NLR be a clinical indicator of disease progression and a robust predictor of all-cause mortality in severe patients (18–20, 38, 39). We herein show high frequency of activated cells in the pool of circulating neutrophils of 3–5 grade CKD patients, and a variable number of neutrophils in the damaged renal tissue. Our unprecedented data, showing CKD conditions disturb neutrophil GPCR expression and the response against infectious stimulus, point out that the imbalance in GPCRs contributes to an aged pool of neutrophils in the blood and an inefficient host defense.

The GPCR CXCR4 is considered the master regulator receptor of neutrophils trafficking in steady–state and host defense, as its expression regulates the bone marrow fate of pre-delivery mature and old homing neutrophils (7). CXCR4 up-regulation was initially observed in aged neutrophils (cultured for 20 hours) before the apoptosis marker Annexin V (AnxV) was expressed. Meanwhile, CXCR4^+^ neutrophils migrate in response to SDF-1, homing to the bone marrow, whereas CXCR4^+^AnxV^+^ cells are apoptotic and unresponsive to SDF-1 (40, 41). Here, CKD neutrophils demonstrated high expression of CXCR4^+^ and showed an increased frequency of CKD neutrophils positive for AnxV, indicating that CKD promotes the aging and death of blood neutrophils.

CXCR4 is widely expressed in several tissues and it plays a key role in modulating the survival, proliferation, and migration of epithelial and endothelial cells (42). In CKD, the overexpression of CXCR4 by epithelial cells has been associated with the development of renal fibrosis, making it a potential target for drug development in CKD (43–46). Hence, these data support our novel findings, highlighting that higher CXCR4 expression by different cells may be involved in CKD.

Activation of the GPCRs FPR1 and FPR2 in neutrophils during infections lead to FPR1-fMLP binding inducing pro-inflammatory actions, while the FPR2 agonist activation, primarily by endogenous mediators, favor efferocytosis and tissue repair (12, 13, 47). Although FPR1 and FPR2 share a high structural and amino acids composition similarity, there is a distinct preference to formylpeptides ligands. A wide range of endogenous and exogenous sources, which act as full or partial agonists, can trigger FPR1 activation. Conversely, FPR2 display a promiscuous nature, binding to a wide range of agonists, including serum amyloid A protein or living bacteria that induces neutrophil migration and the secretion of the pro-inflammatory cytokine CXCL2 (48, 49). This dual function of FPRs in modulating pro-inflammatory or pro-resolutive actions has been elucidated by robust studies that highlight the concept of biased agonism in FPRs. These studies demonstrate that the agonist’s nature and its interaction with the receptor can lead to specific conformational changes, triggering distinct downstream signaling pathways (50–52). Here, we observed that CKD neutrophils express a unique profile of FPRs expressions, characterized by higher expression of both FPR1 and FPR2 receptors. Despite this higher expression, CKD neutrophils were hyporesponsive to FPR1 activation by fMLP.

GPCR activation often leads to the removal of receptors from the cell surface by internalization, and internalized receptors are either recycled to the cell surface or degraded in lysosomes, frequently resulting in downregulation (53). Moreover, GPCRs undergo desensitization and cross-desensitization, which can affect agonist responses. These phenomena have been well established for FPR1-induced chemotaxis. For instance, fMLP-induced chemotaxis occurs over a range of concentrations, and FPR1-fMLP binding desensitizes the C5a receptor, inhibiting neutrophil calcium mobilization and chemotaxis induced by C5a or IL-8 (54–56). The data presented here indicate that CKD may impair GPCR turnover in the cell membrane, resulting in increased expression of CXCR4 and FPRs. Alternatively, FPR1 may undergo cross-desensitization by CKD compounds in the blood, rendering neutrophils unresponsive to specific agonists. Indeed, CKD neutrophils respond differently based on the agonist profile. For instance, studies demonstrated that CKD neutrophils challenged with LPS showed a higher secretion of TNF-α and IL-1β and upregulation, but downregulation of mRNA expression of SOD2 and IL-1a (57, 58). On the other hand, CKD neutrophils were responsive to liposoluble molecules such as n-3 fatty acids and coenzyme Q-10. These molecules reduced the levels of pro-inflammatory leukotrienes and myeloperoxidase and increased the levels of LTB5 (59, 60). Based on these data and findings, it is clear that CKD neutrophils’ responsiveness is dependent on ligands nature. It requires further investigation to better understand how CKD and related disorders disrupt the fine-tuning balance in neutrophil biology.

The mobilization of subsets of intracellular granules to the plasma membrane occurs at the onset of neutrophil responsiveness. Previous data have shown that FPR1 is intracellularly co-localized with CD11b/CD18 and is mobilized to the external surface of the cell to mediate adhesion to the endothelium and subsequent migration into inflamed tissue (61). We also demonstrated that CD11b/CD18 expression is upregulated in CKD neutrophils, indicating an exacerbated ability of neutrophil degranulation in CKD. Although we aimed to address the impaired function of neutrophils in CKD patients, one of the limitations of our study was the small sample size, which did not allow us to categorize how GPCR expression and function are affected by CKD stage progression. Additionally, all subjects in our study were non-dialysis patients undergoing conservative therapies to attenuate base diseases, such as diabetes and higher blood pressure.

Among leukocytes, neutrophils have the highest content of AnxA1, which is secreted, phosphorylated, and displays autocrine, juxtacrine, and paracrine anti-inflammatory properties by binding to FPR2 (12). Glucocorticoids and cytokines stimulate AnxA1 synthesis and release to halt and resolve the acute inflammatory response. In resting conditions, AnxA1 is mainly stored in neutrophil gelatinase granules and rapidly released after neutrophil stimulation using ABC transport or microvesicles (12, 62). Indeed, AnxA1 microvesicles are the major subset released during an inflammatory response (63, 64). Our data show high intracellular content of AnxA1 in CKD neutrophils and low levels of expression at the membrane or secretion into the supernatant, pointing out the inability of CKD neutrophils to release the protein. This inability suggests an inefficient function of neutrophils in the resolution of inflammation, as apoptotic neutrophils express membrane AnxA1 as an “eat me” signal to phagocytic macrophages (65, 66).

The contribution of neutrophils as pro or anti-inflammatory players in renal tissue damage remains unclear. Recently, it was proposed that migrated neutrophils switch to a SiglecF^+^ phenotype due to actions of inflammatory mediators released by damaged epithelial cells. This suggests the presence of a neutrophil subset as pivotal to fibrosis development (67). The analysis of kidney tissue from fibrotic CKD patients revealed a variation in the number of neutrophils among biopsy samples, with some positive for AnxA1. Given previous data indicating the inability of CKD neutrophils to secrete the protein, the contribution of AnxA1 to tissue damage remains to be elucidated. Moreover, AnxA1 is secreted by renal epithelial cells in CKD patients and experimental models of kidney injury; however, the data obtained thus far are controversial (68–71).

## 5 Conclusion

Associated, the data presented herein corroborates the line of evidence suggesting that CKD renders neutrophils unresponsive and indicates that the unbalanced expression of GPCRs at the membrane contributes to the impaired responses in the host defense.

## Data Availability

The raw data supporting the conclusions of this article will be made available by the authors, without undue reservation.
