# Supplementary figures and images for: GPCRs overexpression and impaired fMLP-induced functions in neutrophils from chronic kidney disease patients
Source: Front Immunol. 2024 Aug 26;15:1387566. doi: 10.3389/fimmu.2024.1387566 (PMC11381270; doi:10.3389/fimmu.2024.1387566)

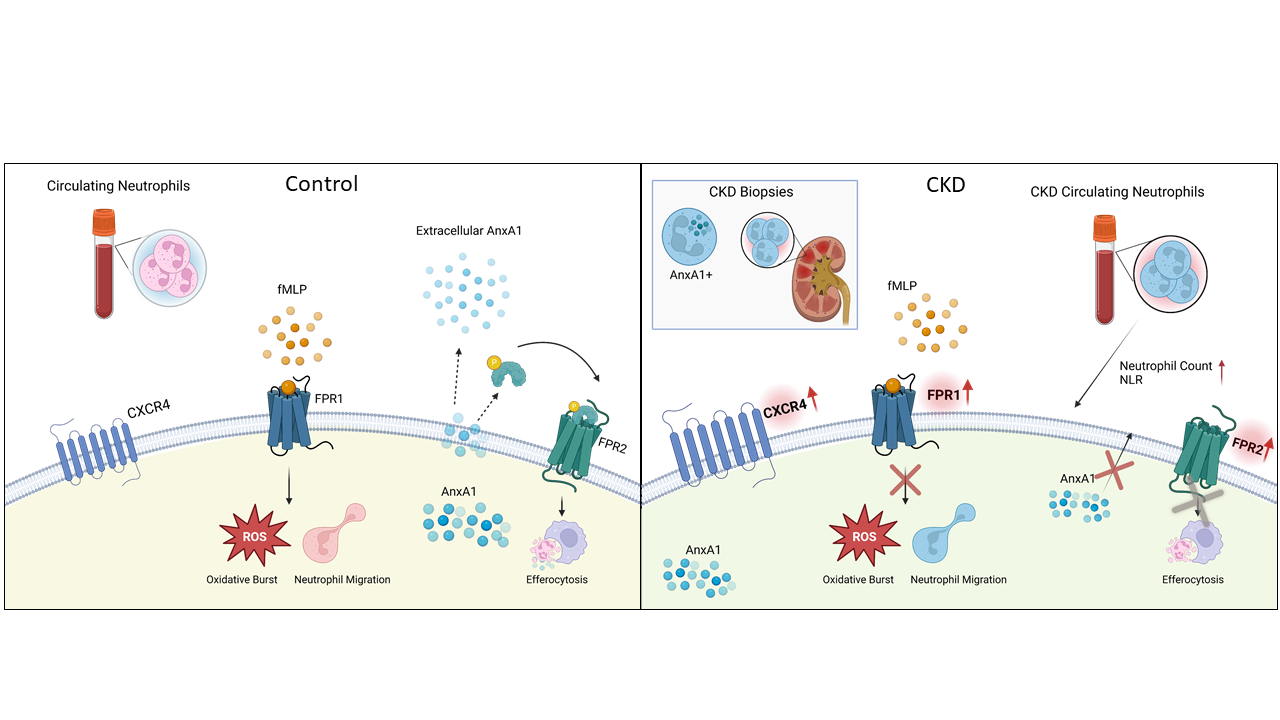

Supplement: Supplementary file 1 [file Image1.tif]

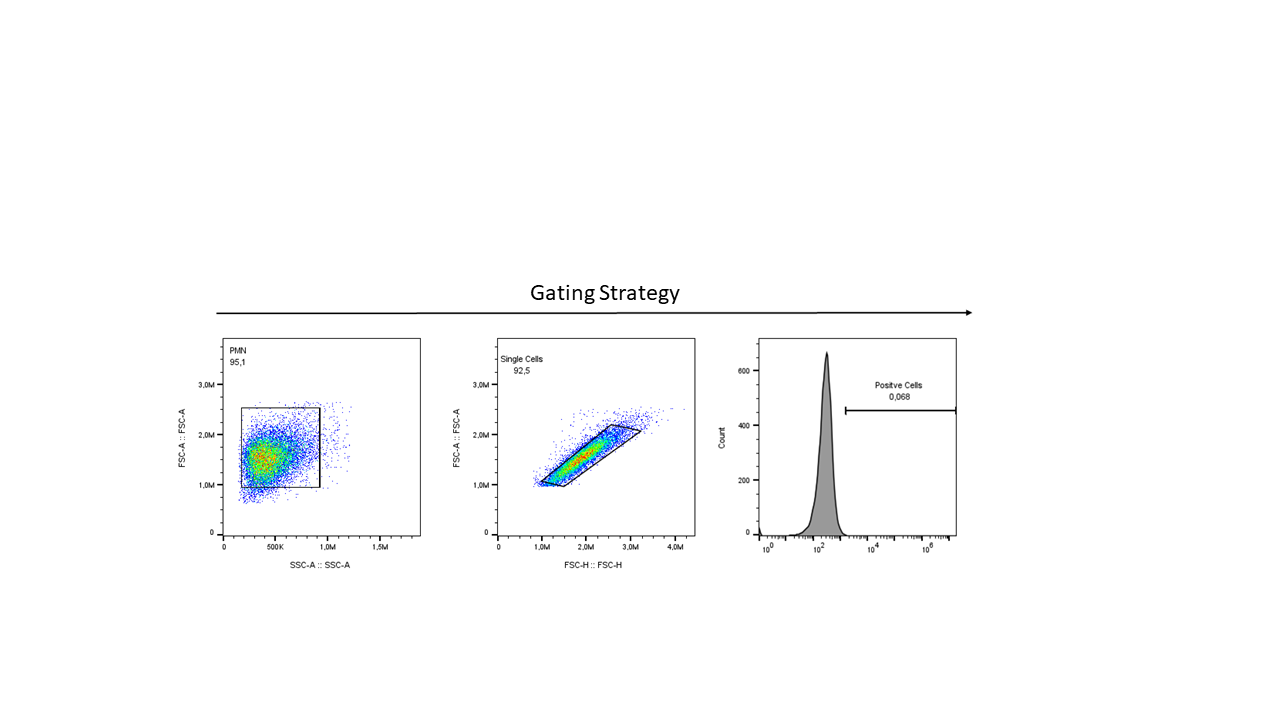

Supplement: Supplementary file 2 [file Image2.png]
