# Supplementary material for: GPCRs overexpression and impaired fMLP-induced functions in neutrophils from chronic kidney disease patients
Source: Front Immunol. 2024 Aug 26;15:1387566. doi: 10.3389/fimmu.2024.1387566 (PMC11381270; doi:10.3389/fimmu.2024.1387566)
Supplement: Supplementary file 3 [file Table1.docx]

Supplementary Material

**Neutrophils overexpressing GPCRs are hyporesponsive to FPR1-mediated signaling in chronic kidney disease patients**

**Pablo Sharf^1†^, Silvana Sandri^1†^, Felipe Rizzetto^1,2†^, Luana Filippi Xavier^1^, Daniela Grosso^3^, Rebeca D. Correia-Silva^4^, Pedro S. Farsky^5^, Cristiane D. Gil ^4^, Sandra Helena Poliseli Farsky^1*^**

^1^Department of Clinical and Toxicological Analyses, School of Pharmaceutical Sciences, University of São Paulo, São Paulo, Brazil

^2^Lagoa Federal Hospital, Rio de Janeiro, Rio de Janeiro, Brazil

^3^ Galileo Biotech, Rio de Janeiro, Rio de Janeiro, Brazil

^4^Department of Morphology and Genetics, Federal University of São Paulo, São Paulo, São Paulo, Brazil

^5^Dante Pazzanese Institute of Cardiology of Sao Paulo, São Paulo, São Paulo, Brazil

**^†^** These authors share first authorship

***Correspondence:**
[sfarsky@usp.br](mailto:sfarsky@usp.br)

# Supplementary Data

## Biopsies data

Detailed information about microscopy of CKD biopsies.

Patient #1 – Female, 65 years old, eGFR 50, Cr 1.2 mg/dL, CKD Stage 3a

Right kidney: Clear cell renal adenocarcinoma with areas of cystic degeneration and tumor necrosis. Ureter and hilar vessels free of malignancy. Absence of tumor infiltration in the renal capsule and perirenal fat. Non-tumorous parenchyma has glomeruli and capillary loops of the usual pattern. Absence of hilar lymph nodes. Free surgical margins. Fuhrman nuclear degree 2; Staging: pT1b

Patient #2 – Female, 63 years old, eGFR 51, Cr 1.2 mg/dL, CKD Stage 3a.

Right kidney: Clear cell renal adenocarcinoma. Presence of tumor infiltration into the perirenal capsule and fat. No tumor necrosis. There was no evidence of vascular and perineural infiltration. There was no evidence of the presence of adrenal glands and lymph nodes. Free surgical margins. Fuhrman nuclear grade: 2; Staging: pT3a.

Patient #3 - Female, 63 years old, eGFR 63, Cr 1. mg/dL, CKD Stage 2.

Right kidney: Papillary-type renal cell carcinoma with chromophilic cells. Located in the upper renal pole. There is no involvement of the adipose tissue of the sinus or renal pelvis. Renal hilum vessels free of neoplasia. Absence of necrosis. Capsule, renal pelvis, perirenal fat, adrenal gland, and ureter free of neoplasia. Free surgical margins. Fuhrman nuclear grade: 2. Staging: pT1b, pNX.

Patient #4 – Male, 56 years old, eGFR 58, Cr 1.5 mg/dL, CKD Stage 3a.

Left kidney: Clear cell renal adenocarcinoma with the presence of areas of necrosis. Absence of tumor filtrate in perirenal fat. Urethral limit and hilar vessels free of neoplasia. Non-neoplastic renal parenchyma without significant histopathological alterations. Fuhrman nuclear grade:3.; Staging: pT1b, pNX.

Patient #5 – Female, 64 years old, eGFR 41, Cr 1.2 mg/dL, CKD Stage 3b.

Right kidney (total nephrectomy): Clear cell renal adenocarcinoma with area of necrosis and hemorrhage. Presence of capsular invasion. Adrenal without abnormalities. Non-isolated lymph nodes. Free surgical margins. Fuhrman nuclear degree 2; Staging: pT3, pNX.

# Supplementary Table 1. Social, demographic, and medicine intake data.

|  | Control | | CKD | |
| --- | --- | --- | --- | --- |
|  | Average | min - max | Average | min – max |
| Age | 38,3 ± 8,14 | 29 - 51 | 62,62 ± 11,35 | 30 – 81 |
| Weight (kg) | 77,88 ± 21,22 | 65 - 90 | 79,68 ± 21,55 | 51 – 119 |
| BMI | 26,12 ± 2,31 | 24,5 - 31,5 | 28,79 ± 5,76 | 22,5 - 44,1 |
|  |  |  |  |  |
|  | **Demographic Data (%)** | | | |
| Alcoholism | 5,55 | | | |
| Smoking | 11,1 | | | |
| Diabetes Mellitus II | 40 | | | |
|  |  |  |  |  |
|  | **Medicine Intake (%)** | | | |
| Statins | 30,5 | | | |
| Vasodilators | 8,3 | | | |
| ACE inhibitors | 5,5 | | | |
| Beta-blockers | 30,5 | | | |
| Fibrates | 30,5 | | | |
| Thiazide Diuretics | 5,5 | | | |
| Dihydropyridines | 13,8 | | | |
| NSAIDs | 11,1 | | | |
| Angiotensin Receptor Blockers | 11,1 | | | |
| Antihyperglycemic | 25 | | | |
| Insulin | 8,3 | | | |
